# Nutrition Care Practices in Colorectal Cancer: A National Survey of Patients, Caregivers, and Healthcare Professionals in Canada

**DOI:** 10.3390/curroncol33090547

**Published:** 2026-09-10

**Authors:** Iris M. Karry, Sally S. Chung, Barry D. Stein, Olga Graifer, Katherine L. Ford, R. Thomas Jagoe, Marianne Fillion, Karmen More, Natasha Bassett-Saltarelli, Joseph Chon, Natalie Leon, Timothy R. Asmis, Martin Chasen

**Affiliations:** 1 Colorectal Cancer Canada, Montreal, QC H3Z 2P9, Canada; sallyc@colorectalcancercanada.com (S.S.C.); barrys@colorectalcancercanada.com (B.D.S.); natashabs@colorectalcancercanada.com (N.B.-S.); 2 Department of Clinical Nutrition, Cedars Cancer Centre, McGill University Health Centre, Montreal, QC H4A 0B1, Canada; olga.graifer@muhc.mcgill.ca; 3 Department of Kinesiology and Health Sciences, University of Waterloo, Waterloo, ON N2L 3G1, Canada; katherine.ford@uwaterloo.ca; 4 Canadian Malnutrition Task Force, Canadian Nutrition Society, Kemptville, ON K0G 1J0, Canada; 5 McGill Cancer Nutrition Rehabilitation Program, Jewish General Hospital, Montreal, QC H3T 1E2, Canada; thomas.jagoe@mcgill.ca; 6 Purdie Pascoe Ltd., London SW11 4LR, UK; marianne.fillion@purdiepascoe.com; 7 Department of Clinical Nutrition, Princess Margaret Cancer Centre, University Health Network, Toronto, ON M5G 2M9, Canada; karmen.more@uhn.ca; 8 Division of Gastroenterology and Hepatology, University of Toronto, Toronto, ON M5S 2W6, Canada; 9 Segal Cancer Center, Jewish General Hospital, Montreal, QC H3T 1E2, Canada; nleon@jgh.mcgill.ca; 10 The Ottawa Hospital Cancer Centre, Ottawa, ON K1H 8L6, Canada; tasmis@toh.ca; 11 Palliative Care, William Osler Health System, Brampton, ON L6R 3J7, Canada; martin.chasen@williamoslerhs.ca; 12 Faculty of Medicine, University of Toronto, Toronto, ON M5S 2W6, Canada; 13 Faculty of Family Medicine, McMaster University, Hamilton, ON L8S 4L8, Canada

**Keywords:** colorectal cancer, nutrition care, patient-centered care, malnutrition, nutritional screening

## Abstract

Nutrition is an essential component of colorectal cancer (CRC) care, influencing treatment tolerance, functional status, and quality of life, yet it remains inconsistently integrated into practice. This study examined experiences with nutrition management and gaps in nutrition care among patients with CRC, caregivers, and healthcare professionals across Canada through a multi-center, cross-sectional survey conducted from July 2025 to February 2026. A total of 243 participants completed the survey, including 121 patients, 45 caregivers, and 77 healthcare professionals. Among patients, 51.2% reported that nutritional status was discussed or assessed during treatment, while 24.0% reported it was never discussed. More than half of patients (55.4%) said they did not receive adequate information regarding weight loss during treatment. Caregivers described substantial nutrition impact symptoms and identified a need for clearer guidance, emotional support, and improved access to dietitians. Healthcare professionals rated nutrition care as highly important yet reported barriers to effective delivery, including limited staffing, lack of standardized screening, and reactive care processes. Across groups, respondents emphasized the importance of timely, personalized, and culturally relevant nutrition care. Overall, nutrition remains insufficiently embedded in CRC care, highlighting the need for routine screening, standardized referral pathways, earlier intervention, and integrated patient-centered nutrition care models to improve CRC care.

## Simple Summary

Nutrition is an important part of colorectal cancer care because it can impact patients’ ability to tolerate treatment, maintain strength, and manage daily life. However, nutrition care is not consistently or proactively integrated into cancer care. In this national Canadian survey, patients with colorectal cancer reported that nutrition was discussed only sometimes or not at all during treatment, and caregivers often felt they lacked clear guidance and support. Healthcare professionals recognized the importance of nutrition but described barriers such as limited staffing, delayed referrals, and lack of routine nutrition risk screening. As a result, nutrition care is often introduced only after challenges have escalated, reflecting a gap in cancer care. These findings highlight the need for standardized processes around earlier screening for malnutrition, clear referral pathways, integrated nutrition assessments, and improved access to consistent, patient-centered nutrition care throughout the colorectal cancer continuum.

## 1. Introduction

Colorectal cancer (CRC) is the fourth most commonly diagnosed cancer in Canada and the second leading cause of cancer-related mortality among men and women combined [1]. Although survival rates for colorectal cancer have improved over time, largely due to organized screening programs and advancements in treatment [1], there has been a notable epidemiological shift toward disease onset at a younger age [2]. In the United States, CRC has recently become the leading cause of cancer-related death among adults younger than 50 years [3].

Nutrition plays a critical role across the cancer care continuum, influencing treatment tolerance, functional status, and overall quality of life [4,5]. Unintentional weight loss in CRC can result from multiple overlapping factors, including inadequate energy and protein intake, cancer-related metabolic changes and symptoms, and treatment-related side effects, leading to depletion of body protein reserves, micronutrient deficiencies, muscle loss, and progressive weight loss [4,5]. Many patients with CRC also experience ostomy-related concerns, altered bowel function, and low anterior resection syndrome (LARS), which can further complicate symptom management, recovery, and weight maintenance [6]. Patients receiving treatment for CRC are particularly vulnerable to nutrition-related decline, with significant unintended weight loss frequently observed in advanced gastrointestinal cancers [7]. These nutrition-related challenges are associated with poorer clinical outcomes, including reduced treatment tolerance, increased toxicity, prolonged hospital stays, and diminished quality of life [5,8]. Notably, an estimated 10–20% of cancer-related deaths are attributed to inadequate nutritional intake rather than the cancer itself [9].

Weight loss and changes in body composition, particularly the loss of skeletal muscle, are increasingly recognized as key drivers of poorer cancer outcomes [10,11]. Although these effects are multifactorial, there is growing evidence that reduced dietary intake is a major and clinically significant contributor, with metabolic alterations also playing a role [12]. These factors underscore the importance of early, proactive nutrition care focused on maintaining adequate dietary intake and preserving muscle mass across all stages of CRC, consistent with international guidelines advocating for routine nutrition screening, assessment, and intervention throughout cancer care [9,13].

The Colorectal Cancer Canada (CCC) Nutrition Program was established to advance policy, education, and practice related to nutrition in the context of CRC in Canada. As part of this initiative, CCC conducted a national cross-sectional survey to capture nutrition care experiences of patients with CRC, their caregivers, and healthcare professionals (HCPs).

## 2. Materials and Methods

### 2.1. Survey Design and Questionnaire

The survey was designed to address two primary objectives: (1) to explore the approaches to, and experiences of, nutrition management among patients, caregivers, and HCPs during CRC treatment; and (2) to explore participants’ perceptions of the availability, accessibility, and adequacy of nutrition care resources in Canada.

In alignment with these objectives, the survey aimed to generate two key outcomes: (1) insight into the perspectives and experiences of participants related to nutrition management following a CRC diagnosis; and (2) evidence regarding participants’ perceived availability, accessibility, and adequacy of nutrition care resources in Canada.

Respondents were directed to a tailored set of questions based on their self-identified respondent group. The expected completion time was approximately 20 min, and the survey was available in both English and French. The survey was developed by members of CCC to address the study objectives and capture both quantitative and qualitative data on nutrition care across the CRC care continuum. The patient and HCP questionnaires each comprised 26 items, including 4 open-ended questions, and the caregiver questionnaire comprised 23 items, including 4 open-ended questions. The open-ended questions were included to provide contextual and explanatory depth to the quantitative survey findings. Draft versions of the questionnaire were reviewed and refined by CCC’s Nutrition Program Expert Advisory Committee, composed of HCPs and researchers, to ensure clarity, relevance, and face validity. The full survey instrument is provided in Supplementary Material S1.

### 2.2. Study Population and Participant Recruitment

The target population included people in Canada who had been diagnosed with CRC, caregivers of individuals with CRC, and HCPs involved in the treatment of patients with CRC in Canada. Respondents were eligible to participate if they were 18 years of age or older and met one of the following criteria: (1) *patients* diagnosed with any stage of CRC within the past 5 years who had undergone or were currently undergoing treatment; (2) *caregivers* of patients diagnosed with CRC within the past 5 years who had undergone or were currently undergoing treatment; or (3) *HCPs* currently treating patients with CRC.

Participants were recruited using a convenience sampling approach through a multi-center recruitment strategy. Recruitment materials, including social media posts, a study poster, and a Letter of Invitation, were distributed through CCC’s established communication platforms including social media accounts, monthly newsletters, email distribution lists of HCPs and advisory committee members, public health units, community partners, community health centres, and academic hospitals across Canada. Additional participants were reached through snowball sampling and word-of-mouth.

**Figure 1:**
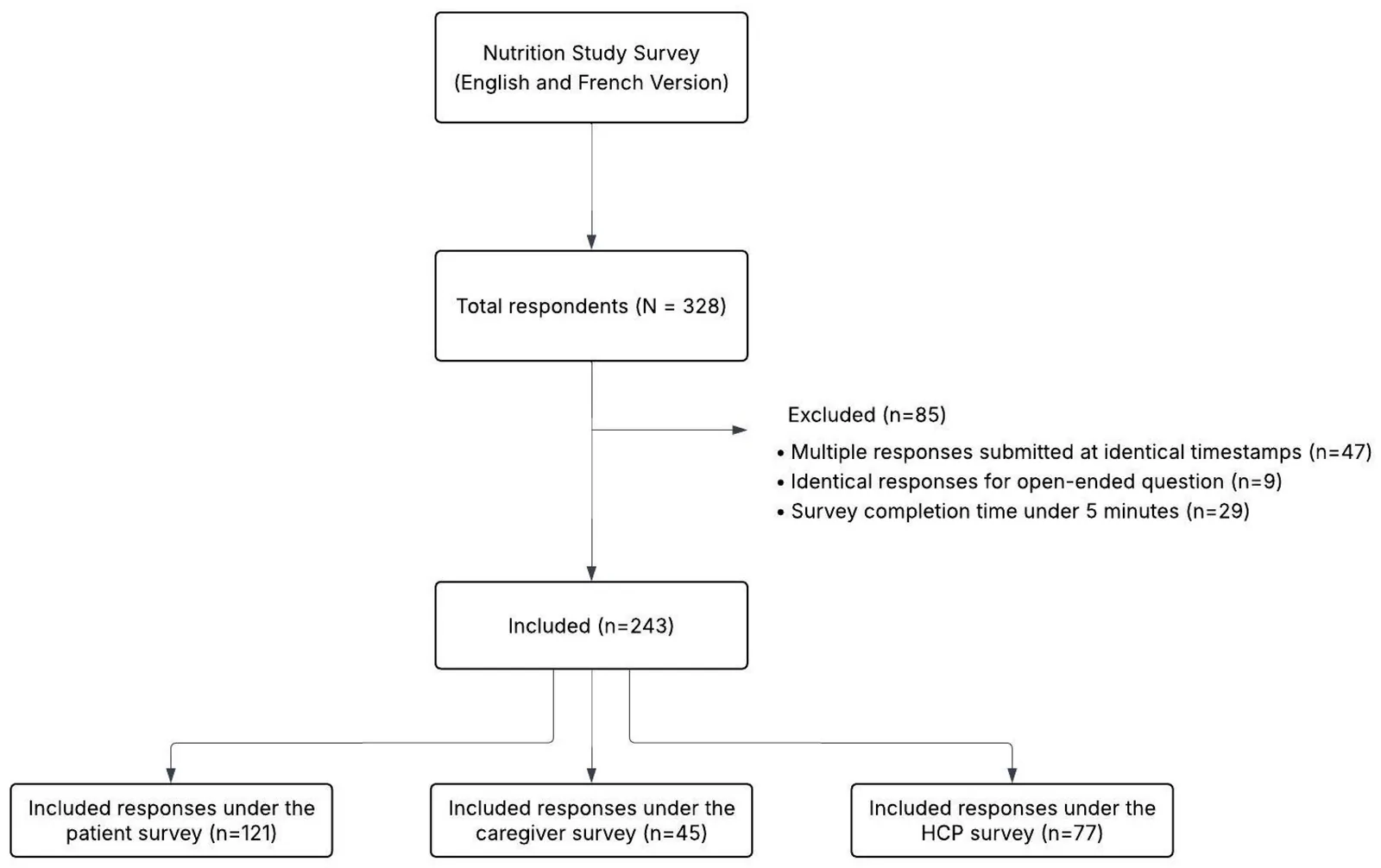
Flowchart of survey respondents included in the study.

Responses were excluded if they met one or more of the following criteria: duplicate submissions (e.g., multiple entries submitted at the same timestamp), identical word-for-word responses to open-ended questions suggesting non-genuine participation, or survey completion times of less than 5 min, indicative of insufficient engagement with the survey content (Figure 1).

A total of 328 responses were received. Following application of the exclusion criteria, 243 responses were retained for analysis, including 121 patient responses, 45 caregiver responses, and 77 HCP responses.

### 2.3. Data Collection

Data were collected over a seven-month period, from 4 July 2025 to 9 February 2026. The survey was administered through an online survey platform hosted by Purdie Pascoe (London, UK). Participants accessed the survey through a survey link or QR code.

### 2.4. Data Analysis

243 eligible responses were retained for analysis. Quantitative data were analyzed using descriptive statistical methods. Categorical variables were summarized using frequencies (*n*) and percentages (%), calculated within each respondent category. All quantitative analyses were conducted using *Microsoft Excel (version 16.0)*. The qualitative portion of the study included analysis of the open-ended questions. Open-ended responses were analyzed using thematic analysis [14] to identify recurring patterns and key themes across participants’ responses. Responses were reviewed and coded by two members of the research team (S.C., I.M.K.), with similar codes grouped into broader themes. Coding and thematic development were conducted separately for patients, caregivers, and HCPs to preserve group-specific perspectives. Themes were developed by examining relationships and recurring patterns among the codes and were subsequently reviewed against the original responses to ensure that the themes remained grounded in the data. Themes were subsequently compared across groups to identify shared patterns and divergent experiences.

### 2.5. Ethics and Consent

Ethics approval was obtained from the William Osler Health System Research Ethics Board on 13 May 2025 (Osler REB number 0257). Informed consent was obtained electronically, and all survey responses were collected anonymously. Participants had the option to enter a gift card draw and/or receive a summary of study findings by providing their email address through a separate survey link. To maintain anonymity, email addresses were not linked to survey responses and were stored separately from the study data.

## 3. Results

### 3.1. Respondent Characteristics

A total of 243 respondents participated in the study, including 121 patients diagnosed with CRC, 45 caregivers, and 77 HCPs (Figure 1). Among patient respondents, 83 (68.6%) were between 31 and 60 years of age, with the largest age groups being 31–40 years (*n* = 32, 26.4%) and 41–50 years (*n* = 30, 24.8%). Most patients resided in population centres, with 53.7% (*n* = 65) living in large urban population centres, 28.1% (*n* = 34) in medium population centres, and 13.2% (*n* = 16) in small population centres. Only 4.1% (*n* = 5) of patients resided in rural areas. Most patients reported a diagnosis of rectal cancer (*n* = 83, 68.6%), while 31.4% (*n* = 38) reported colon cancer. A range of disease stages was represented, with stage II (*n* = 33, 27.3%), stage IV (*n* = 25, 20.7%), and stage III (*n* = 24, 19.8%) reported most frequently. Most patients received chemotherapy (*n* = 88, 72.7%) and surgery (*n* = 80, 66.1%), with just over a third having received radiation (*n* = 43, 35.5%). Caregivers were most commonly supporting a spouse, partner, or friend (*n* = 24, 53.4%), often for durations exceeding 6 months (*n* = 36, 80%). HCP respondents represented a multidisciplinary group, including registered dietitians (*n* = 16, 20.8%), nutritionists (*n* = 14, 18.2%), and nurse navigators (*n* = 13, 16.9%), and primarily practiced in hospital-based settings (*n* = 68, 88.3%). The three respondent groups were geographically distributed across Canada, with the largest proportions residing in Ontario, British Columbia, and Alberta. Detailed demographic characteristics are provided in Supplementary Tables S1–S3.

### 3.2. Nutrition Impact Symptoms Across Groups

Patients most frequently reported loss of appetite, fatigue, nausea, and diarrhea during cancer treatment, with similar patterns reported by caregivers and HCPs (Table 1). Pain and additional gastrointestinal symptoms, including vomiting, constipation, early satiety, and taste changes, were also frequently reported across groups.

Beyond symptom prevalence, respondents highlighted the functional and nutritional consequences of these challenges. Among patients, 66.1% (*n* = 80) reported concerns about inadequate intake, and 52.9% (*n* = 64) indicated that weight loss interfered with their ability to carry out usual activities. Caregivers similarly reported high levels of concern related to inadequate intake (*n* = 33, 73.3%) and functional decline (*n* = 33, 73.3%) among those they supported, with 62.2% (*n* = 28) indicating that weight loss felt uncontrolled.

### 3.3. Gaps in Nutrition Care Delivery and Timing Across the CRC Care Continuum

Despite the high burden of nutrition impact symptoms reported across all respondent groups (Table 1), respondents described nutrition care as not consistently or proactively integrated into CRC management. Among patients, just over half (*n* = 62, 51.2%) reported that their nutritional status was discussed or assessed throughout treatment, while nearly one-quarter (*n* = 29, 24.0%) indicated that it was never addressed. More than half (*n* = 67, 55.4%) also reported not receiving adequate information regarding weight loss during treatment.

Caregiver responses reflected similar gaps in nutrition-related guidance and support. Although caregivers frequently managed nutrition-related challenges (Table 1), only one-third (*n* = 15, 33.3%) reported that the individual they supported received adequate information about weight loss. In open-ended responses, caregivers emphasized the need for clearer, more practical guidance and greater involvement in nutrition-related discussions, particularly given their central role in meal preparation and day-to-day care.

HCPs, while recognizing the importance of nutrition care, identified structural and process-related barriers that limit its consistent delivery. Over three-quarters (*n* = 59, 76.6%) rated nutrition care as very or extremely important; however, care models were often characterized as reactive rather than proactive. Key barriers identified in open-ended responses included limited dietitian availability, high clinical workload, and the absence of standardized screening and referral pathways. Respondents also indicated that nutrition is not routinely embedded into care pathways, with one stating that “the care plan is not structured around nutritional needs,” and another noting that screening often occurs only after treatment initiation rather than earlier in the disease trajectory.

### 3.4. Access to Nutrition Care, Information, and Barriers

Access to nutrition care and information varied across the care continuum, with respondents describing reliance on a combination of formal services and informal or self-directed strategies. Among patients, about one third reported consulting a provincially regulated nutrition professional (dietitian or nutritionist, depending on the province), while most relied on informal supports such as family and friends for nutrition care and online resources, including websites and forums (Table 2). Caregivers reported a similar pattern, combining clinical guidance, most commonly from dietitians, with information obtained through digital platforms and peer networks (Table 3). While these informal resources were perceived as helpful and accessible by patients and caregivers (Supplementary Tables S4 and S5), their use occurred alongside reported variability in the availability and consistency of formal nutrition services. HCPs acknowledged the value of peer and community-based support but raised concerns about the quality and reliability of information, noting that inconsistent or inaccurate guidance could complicate care and influence patient decision-making. One healthcare professional explained, “often times the patients have done their own ‘Doctor Googling’ and we have to do damage control...”.

Despite the availability of nutrition services within many clinical settings, timely and sustained access was inconsistent. Patients reported variable wait times for dietitian consultations after diagnosis, with the largest proportion (*n* = 36, 29.8%) reporting a wait time between 2 and 4 weeks, with some (*n* = 13, 10.7%) experiencing delays exceeding two months. Caregivers similarly described variability in access across institutions and emphasized that early nutrition care was not consistently offered as a standard component of care. In contrast, nearly half (*n* = 34, 44.2%) of HCPs reported that patients could access a dietitian within one week of referral. Open-ended responses from HCPs, however, highlighted that limited staffing, high workload, and prioritization of higher-acuity patients often restricted ongoing access and follow-up care.

In open-ended responses, patients described receiving general or insufficiently tailored guidance and difficulty navigating nutrition information, with one patient noting that “there’s a lot of contradicting information about what’s good and bad for you.” HCPs identified challenges related to patient health literacy and perceptions surrounding nutrition care that may affect referral uptake and engagement with care. As one healthcare professional explained, “Some patients just ‘don’t know what they don’t know’ and refuse a referral,” while some family members may perceive a patient’s weight loss as their own personal failure and the need for nutrition care as “a personal attack”, contributing to resistance toward seeking additional help.

### 3.5. Perceived Relevance and Personalization of Nutrition Care

Patients and caregivers emphasized the importance of personalized, practical, and culturally relevant nutrition guidance. Among patients, 57.9% (*n* = 70) indicated that it was very or extremely important that nutritional information reflected their cultural background and dietary preferences. However, alignment between recommendations and individual preferences was variable, with many patients reporting that guidance was only somewhat tailored to their needs.

HCPs similarly reported mixed perceptions regarding the availability and relevance of nutrition resources. While some (*n* = 25, 32.5%) rated existing resources positively, others (*n* = 24, 31.2%) indicated that current resources do not consistently reflect the cultural diversity or dietary preferences of the populations they serve.

### 3.6. Themes from Open-Ended Survey Responses

Open-ended survey responses provided additional context to the quantitative findings and highlighted perceived barriers, unmet needs, and opportunities to improve nutrition care in CRC care. Five overarching themes were identified: (1) multidisciplinary and collaborative care, (2) individualized and personalized nutrition planning, (3) education and engagement, (4) accessibility and continuity of care, and (5) structural capacity and infrastructure. Table 4 summarizes these themes with representative quotations across respondent groups.

## 4. Discussion

This study provides a multi-stakeholder perspective on nutrition care in CRC, drawing on the experiences of patients, caregivers, and HCPs across Canada. The findings highlight important gaps in the delivery of nutrition care and identify opportunities to strengthen its integration within CRC management, reinforcing the need to recognize nutrition as an integral component of CRC care rather than a secondary or supportive consideration.

A central issue identified in this study is the challenge of providing timely and adequate nutrition care across the CRC trajectory. Although nutrition-related concerns were commonly reported among all three respondent groups, patients and caregivers described limited discussion of nutrition with HCPs and insufficient guidance on managing treatment-related weight changes. While HCPs generally perceived access to dietitian services as relatively prompt following referral, patients’ experiences suggested that delays and inconsistencies remain. This discrepancy points to a gap not only in access but in how nutrition care is experienced and delivered, indicating that the presence of services alone may not translate into meaningful or timely support from the patient’s perspective. These findings align with current literature indicating that dietary consultation is not consistently integrated into CRC care despite guideline recommendations, reflecting a broader policy-practice gap in the implementation of nutrition care [15,16].

Survey findings also underscore the multidimensional nature of nutrition-related burden in CRC. Across respondent groups, nutrition concerns were rarely experienced in isolation; rather, multiple interacting symptoms disrupted daily functioning and hindered adequate dietary intake. Fatigue emerged as a particularly important barrier, limiting patients’ ability to plan, prepare, and consume meals, while caregivers and HCPs emphasized how ongoing weight loss and functional decline further compounded difficulties in maintaining nutritional intake. These findings reinforce that nutritional risk in CRC extends beyond food intake or weight change alone, highlighting the importance of nutrition screening and assessment approaches that also consider symptom burden, functional status, and psychosocial factors. These findings are consistent with prior research demonstrating that nutritional risk in CRC is shaped by overlapping physical, treatment-related, and contextual factors [17,18]. As such, effective nutrition care may require coordinated, multidisciplinary approaches that incorporate psychological support, symptom management, and physical rehabilitation alongside dietary counselling.

Despite identifying both the importance of nutrition and the complexity of nutrition-related challenges in CRC, respondents described nutrition care delivery as misaligned with patient needs, with care often initiated after concerns emerged rather than integrated early into the disease trajectory. HCPs highlighted system-level barriers such as insufficient dietitian staffing to allow for timely referral and regular follow-up, inconsistent nutrition screening, and care plans structured around management of acute symptoms rather than nutritional needs. Although an in-depth analysis of these system-level factors was beyond the scope of this study, together they point to a care environment in which nutrition care is not adequately embedded in routine oncology practice, particularly at critical time points such as diagnosis and treatment initiation, when nutrition care may have the greatest impact [19,20]. This delay in accessing nutrition care may be particularly consequential in CRC. Many patient and caregiver respondents expressed concern about weight loss, which can develop progressively throughout treatment alongside declines in muscle mass and strength, adversely affecting treatment tolerance and patient outcomes [10,11,21]. Similarly, HCP respondents described a mismatch between care structures and the “dynamic nature of the disease”, with symptoms, disease status, and treatment needs shifting continuously. Limited nutrition training in medical education may further compound these challenges, contributing to inconsistent nutrition-related knowledge and messaging across the care team [22,23].

Amid these gaps, patients and caregivers often take a central role in managing nutrition-related challenges. All three respondent groups emphasized the need for more personalized, practical, and culturally relevant nutritional guidance that reflects individual symptoms, treatment stage, comorbidities, and dietary preferences. Existing research highlights the importance of individualized nutrition care that evolves over the course of treatment and recovery, underscoring the need for ongoing follow-up and adaptation rather than a one-time intervention [4,5,8,9]. However, when such support is limited, patients and caregivers may rely on self-directed strategies and external sources of information, placing greater responsibility on individuals to navigate often inconsistent nutrition advice. This finding is consistent with a scoping review by Abedin et al. which identified limited evidence for nutrition self-management interventions and gaps in online resources and clinical guidelines, leaving many patients without timely, trustworthy, and practical dietary guidance throughout CRC care [24]. 

Taken together, these findings highlight the need for earlier and consistent integration of nutrition care throughout the CRC continuum. Addressing the gaps identified by patients, caregivers, and HCPs in this study will likely require coordinated action across multiple levels of the care system, such as implementation of standardized nutrition screening and referral processes into CRC care pathways, and the adoption of care models that can be tailored to patients’ changing symptoms and needs over time. Table 5 summarizes potential future research and implementation initiatives identified through this study.

This study has several strengths. It incorporates perspectives from patients, caregivers, and HCPs, enabling a comprehensive, multi-stakeholder understanding of nutrition care in CRC. The national scope of the survey enhances the relevance of the findings across diverse care settings in Canada, and the integration of quantitative and qualitative data provides both breadth and depth in capturing experiences and care gaps.

Several limitations to this study should also be considered. First, the use of non-probability convenience sampling approach may have introduced selection bias and may limit generalizability of the findings. Participants were primarily recruited through CCC communication channels and affiliated networks, which may have resulted in a sample that is more engaged and connected to support services than the broader CRC population. Moreover, individuals engaged through CCC platforms may also demonstrate greater health-seeking behaviors than those who are less connected to patient advocacy networks, further contributing to sampling bias. As these respondents may also have had comparatively greater access to healthcare and nutrition resources, the nutrition care gaps identified in the study may be underestimated. The true extent of unmet nutrition needs among the broader CRC population, including individuals with less engagement or access to supportive care networks, may be greater than reported.

The patient sample was disproportionately composed of individuals diagnosed with rectal cancer and participants from larger provinces (e.g., British Columbia, Ontario, and Alberta) and urban settings, which may limit transferability to rural or underserved populations. Additional limitations include the absence of respondents from Quebec, despite it being a large province, as well as the predominance of nutrition-specialized healthcare professionals and respondents from academic centres, which may have introduced specialty- and resource-related biases.

The survey did not comprehensively capture clinical disease characteristics that may influence nutrition-related experiences. Although information regarding treatments received was collected and is presented in the results, detailed clinical variables such as treatment intent, neoadjuvant versus adjuvant treatment, ostomy status, and low anterior resection syndrome were not systematically collected. Consequently, we were unable to examine how nutrition-related experiences varied across specific disease stages or treatment trajectories. Future studies incorporating clinical data and more detailed treatment and disease characteristics would help clarify nutrition needs within specific colorectal cancer subgroups.

Exploratory subgroup analyses by cancer type and disease stage were conducted but were limited due to the study’s small sample size. Similarly, the sample size was unevenly distributed across patients, caregivers, and HCPs which limited formal statistical comparisons between groups. Accordingly, any observed patterns across groups should be interpreted with caution and confirmed in future studies.

Additionally, participation required time, internet access, and the capacity to complete an online survey. Patients and caregivers experiencing more advanced disease stages, greater symptom burden, cognitive strain, or more severe treatment-related side effects may have been less able or less inclined to participate. Consequently, the perspectives from these individual groups may be underrepresented in the sample.

Finally, the survey relied on self-reported data, which may introduce recall bias. Perceptions of nutritional counselling, resource accessibility, and symptom severity may be influenced by time since diagnosis, emotional experiences, or changing health status.

Future research would benefit from broader recruitment strategies to improve representativeness across disease stages, geographic regions, and levels of resource access. Longitudinal designs, integration of clinical and nutrition status data, and combining patient-reported experience with objective nutrition assessment may also strengthen the applicability of findings.

## 5. Conclusions

This study provides an overview of nutrition-related experiences among patients with CRC, their caregivers, and HCPs across Canada. Despite widespread recognition of the importance of nutrition in CRC care, respondents described current approaches as reactive and inconsistently delivered, with nutrition care perceived as difficult to access in a timely and personalized manner. These findings highlight persistent gaps in the delivery of nutrition care, as experienced by patients, caregivers, and HCPs, and underscore the need for more proactive, coordinated approaches to supporting patients throughout their cancer journey.

Moving forward, the priority should shift from generating evidence alone to ensuring that existing knowledge is translated into practice. Early nutrition intervention and consistent follow-up are widely recognized as important components of CRC care, yet respondents in this study identified persistent logistical and system-level barriers limiting their implementation. Addressing these barriers will require action at both systemic and local levels, including increased government funding to expand dietitian capacity and the implementation of care processes that support proactive nutrition care throughout the disease trajectory.

## Figures and Tables

**Table 1:** Summary of Nutrition Impact Symptoms Reported Across Respondent Groups.

Symptoms	Patients (*n* = 121)	Caregivers (*n* = 45)	HCPs (*n* = 77)
	*n* (%)	*n* (%)	*n* (%)
Loss of appetite	70 (57.9)	27 (60.0)	42 (54.6)
Fatigue	63 (52.1)	23 (51.1)	34 (44.2)
Nausea	58 (47.9)	20 (44.4)	34 (44.2)
Diarrhea	51 (42.2)	17 (37.8)	38 (49.4)
Pain	33 (27.3)	20 (44.4)	36 (46.8)
Vomiting	38 (31.4)	16 (35.6)	36 (46.8)
Constipation	39 (32.2)	16 (35.6)	33 (42.9)
Indigestion	32 (26.5)	16 (35.6)	34 (44.2)
Feeling full too quickly after beginning to eat	36 (29.8)	14 (31.1)	33 (42.9)
Changes to how food tastes and smells	40 (33.1)	13 (28.9)	30 (39.0)
Heartburn	30 (24.8)	9 (20.0)	25 (32.5)
Problems swallowing	19 (15.7)	14 (31.1)	23 (30.0)
Dry mouth	25 (20.7)	14 (31.1)	14 (18.2)
Mouth sores	19 (15.7)	13 (28.9)	19 (24.7)
Other	4 (3.3)	0 (0.0)	1 (1.3)
None of the above	5 (4.1)	0 (0.0)	0 (0.0)

*n*: sample size.

**Table 2:** Patient-Reported Nutrition Impact Symptom Management Strategies (*n* = 121).

Variable	*n* (%)
Support from family or friends	49 (40.5)
Nutritional supplements/meal replacements	47 (38.8)
Consultation with provincially regulated nutrition professional	42 (34.7)
Consultation of websites (e.g., Canadian Cancer Society)	39 (32.2)
Consultation of online communities/forums	37 (30.6)
Physical rehabilitation or physical therapy/exercise	26 (21.5)
Consultation with an endocrinologist	24 (19.8)
Dietary changes	24 (19.8)
Meditation and mindfulness	24 (19.8)
Consultation of scientific journals	23 (19.0)
Support groups	21 (17.4)
Medication	20 (16.5)
Consultation with non-provincially regulated nutrition professional	19 (15.7)
Consultation with a naturopath	19 (15.7)
Consultation of social media (e.g., influencers)	16 (13.2)
Psychological counselling	16 (13.2)
Consultation with other healthcare professionals	14 (11.6)
Consultation of healthcare authorities (e.g., Health Canada)	14 (11.6)
Consultation with a palliative/supportive care specialist	12 (9.9)
No special measures were taken	7 (5.8)
Other	7 (5.8)
Do/did not experience nutritional symptoms	6 (5.0)

*n*: sample size.

**Table 3:** Caregiver-Reported Nutrition Impact Symptom Management Strategies (*n* = 45).

Variable	*n* (%)
Nutritional supplements/meal replacements	17 (37.8)
Support from family or friends	16 (35.6)
Consultation with provincially regulated nutrition professional	13 (28.9)
Consultation with a palliative/supportive care specialist	12 (26.7)
Consultation of websites (e.g., Canadian Cancer Society)	12 (26.7)
Consultation of online communities/forums	11 (24.4)
Physical rehabilitation or physical therapy/exercise	11 (24.4)
Consultation with an endocrinologist	10 (22.2)
Consultation of social media (e.g., influencers)	10 (22.2)
Consultation with non-provincially regulated nutrition professional	9 (20.0)
Consultation of healthcare authorities (e.g., Health Canada)	9 (20.0)
Psychological counselling	9 (20.0)
Support groups	9 (20.0)
Consultation of scientific journals	8 (17.8)
Consultation with a naturopath	6 (13.3)
Dietary changes	5 (11.1)
Medication	3 (6.7)
Meditation and mindfulness	3 (6.7)
Do/did not experience nutritional symptoms	3 (6.7)
Can/could not manage the nutritional symptoms	1 (2.2)
No special measures were taken	1 (2.2)

*n*: sample size.

**Table 4:** Summary of Open-ended Survey Responses with Representative Quotes.

Theme	Description	Quotations
Multidisciplinary and collaborative care	Respondents emphasized the importance of integrated, team-based care models, including embedding dietitians within oncology teams and strengthening coordination across clinical services. Responses highlighted gaps in communication and collaboration across care settings.	• *“A nutritionist or dietitian should be assigned to your care team by default.” **(Patient)***
		• *“Lack of multidisciplinary collaboration—communication gaps among surgery, ICU, nutrition departments, etc., often cause treatment plans to be interrupted during transfers or discharge.” **(HCP)***
Individualized and personalized nutrition planning	Respondents emphasized the need for individualized nutrition care tailored to symptoms, treatment stage, and evolving clinical needs, including adaptable nutrition plans and ongoing follow-up.	• *“On the patient side, efforts can focus on personalized and participatory nutritional support. On one hand, dietitians should develop “one person, one plan” nutrition programs based on the patient’s disease condition, metabolic characteristics, and dietary preferences.” **(Caregiver)***
		• *“More dietitians [are] needed for faster access and more regular follow-up given the dynamic nature of the disease (symptoms change, disease status changes, treatments change etc.) which requires changes in the nutritional recommendations to best suit the patients needs in that moment and increase chances of a better outcome.” **(HCP)***
Education and engagement	Respondents identified gaps in nutrition-related education and engagement, including limited nutrition literacy among patients and caregivers, variable nutrition training among healthcare professionals, and inconsistent awareness of available nutrition services. Responses highlighted the use of practical and accessible educational formats, including digital tools, to support learning and engagement.	• *“Use illustrated manuals, short videos, etc., to teach patients and their families how to read food labels and master simple cooking techniques, so that daily meals better meet treatment needs.” **(Caregiver)***
		• *“I feel that having a brochure regarding what services the dieticians provide and areas they can assist with, as well as any additional supports available, would be very helpful for patients. This would help clear up any misconceptions regarding the role of the dietician as an HCP and perhaps patients would be more open to reach out.” **(HCP)***
		• “*Some patients also just ‘don’t know what they don’t know’ and refuse a referral. Some family members view it as a ‘personal attack’ if a patient is experiencing weight loss, as they feel they are not doing a good enough job and don’t want to ‘need help’ with eating.*” ***(HCP)***
Accessibility and continuity of care	Respondents identified gaps in timely, consistent, and sustained access to nutrition services across the CRC care continuum. These included delays in accessing dietitian support, limited early integration of nutrition care at diagnosis and treatment initiation, and challenges maintaining support beyond acute care settings. Participants highlighted the need for earlier engagement and more continuous nutrition care, including transitions to home- and community-based care.	• *“I had a challenging start to chemo which led to weight loss and a dietician’s involvement would have been welcome at that earlier point and certainly post-surgery recovery. Receiving advice of what to eat or not eat post-surgery would have been helpful.” **(Patient)***
		• *“Insufficient awareness and assessment-healthcare providers often do not prioritize nutritional risk screening, leading to missed diagnoses of high-risk patients due to delayed or absent evaluation upon admission” (**HCP)***
Structural capacity and infrastructure	Respondents identified system-level constraints affecting the delivery of nutrition care, including limited dietitian capacity, funding limitations, and the absence of standardized nutrition screening processes. These structural challenges were described as contributing to delays in access, limited follow-up capacity, and inconsistent delivery of nutrition care.	• *“Limited resources and staffing—insufficient clinical dietitians and untimely supply of enteral/parenteral nutrition products or equipment make it difficult to meet clinical needs.” **(HCP)***
		• *“There’s multiple barriers, but one of them that’s worth emphasizing is the fact that the care plan is not structure around nutritional needs. It’s structured around acute symptoms management.” **(HCP)***
		• “*No official screening happening earlier in their trajectory, mostly only once at the treatment center. Screening should occur more systematically at oncologist visits.*” ***(HCP)***

**Table 5:** Perceived Gaps and Corresponding Areas for Future Studies.

Gap	Research Priorities	Implementation/Quality Improvement Priorities
Delayed delivery of nutrition care across the CRC trajectory	Evaluate the optimal timing and models of nutrition care across the CRC continuum.	Implement standardized nutrition screening using validated tools (e.g., the Patient-Generated Subjective Global Assessment Short Form [PG-SGA-SF], and the Malnutrition Screening Tool [MST]) within institutional supportive care pathways and electronic health records.
		Evaluate the impact of this integration on clinical outcomes and patient-reported experiences of care
Limited personalization of nutrition care for patients with CRC	Investigate approaches to tailoring nutrition care decision-making based on patient factors (e.g., structured approaches to personalizing care based on symptom burden, treatment stage, comorbidities, cultural context, and dietary preferences) and evaluate their feasibility and impact on outcomes	Evaluate the implementation of standardized nutrition screening using validated tools (e.g., MST and PG-SGA-SF) within existing oncology quality improvement initiatives, such as the Quality Oncology Practice Initiative (QOPI) [25] to support earlier identification of nutrition needs and more personalized nutrition care. Emerging models such as the MyPath Nutrition Care Pathway program may also offer useful approaches for strengthening practical implementation [26].
	Investigate subgroup differences (e.g., colon vs. rectal cancer) to inform more targeted and responsive nutrition care approaches.	Evaluate the implementation of culturally and linguistically tailored nutrition resources that incorporate culturally relevant foods and dietary practices to improve patient engagement and nutrition outcomes.
Complex and multifaceted burden of nutrition impact symptoms	Assess the effectiveness of multidisciplinary care models that coordinate nutrition care with symptom management and functional support across the CRC continuum.	
System-level capacity constraints	Evaluate the availability, distribution and utilization of nutrition care resources, including dietitian workforce capacity.	
	Explore alternative delivery models (e.g., virtual care, group-based interventions, digital tools) to improve reach and continuity of care.	
Barriers related to health literacy and misinformation	Examine the prevalence and impact of nutrition-related misconceptions among patients and caregivers	Develop, implement, and evaluate accessible, evidence-based educational and skills-building interventions to improve nutrition literacy.
Limited integration of nutrition into HCP medical education		Develop, implement, and evaluate continuing medical education initiatives to improve non-dietitian HCPs’ knowledge, confidence, and ability to integrate appropriate nutrition information into CRC care

## Data Availability

The data used in this study are not publicly available but may be available upon request. Please contact the corresponding author.

## Supplementary Materials

The following supporting information can be downloaded at https://www.mdpi.com/article/10.3390/curroncol33090547/s1: Supplementary Material S1: Survey Form; Table S1: Patient Respondent Demographic; Table S2: Caregiver Respondent Demographic; Table S3: Healthcare Professional Respondent Demographic; Table S4: Patient Perceived Effectiveness of Nutrition Support and Information and Unmet Needs in Managing Nutrition Impact Symptoms; Table S5: Caregiver Perceived Effectiveness of Nutrition Support and Information and Unmet Needs in Managing Nutrition Impact Symptoms.

## Abbreviations

The following abbreviations are used in this manuscript:

CRC	Colorectal cancer
HCP	Healthcare professional
CCC	Colorectal Cancer Canada
