# Supplementary material for: Nutrition Care Practices in Colorectal Cancer: A National Survey of Patients, Caregivers, and Healthcare Professionals in Canada
Source: Curr Oncol. 2026 Sep 10;33(9):547. doi: 10.3390/curroncol33090547 (PMC13605743; doi:10.3390/curroncol33090547)
Supplement: Supplementary file 1 [file curroncol-33-00547-s001.zip › Supplementary Material 1_CRC Nutrition Survey Questionnaire.pdf]

Exploring Clinical Nutritional Support Experiences of Colorectal Cancer Patients, Caregivers and Healthcare Professionals in Canada

Introduction page

Please choose your preferred language to complete the survey.

- English
- French

Veuillez choisir votre langue préférée pour répondre à l'enquête.

- Français
- Anglais

Are you a:

- Patient
- Caregiver
- Healthcare Professional
- Other (please specify):

Do you currently reside in Canada?

- Yes
- No

Pre-Survey Screening Questions for Patients

Are you 18 years of age or older?

- Yes
- No

Have you been diagnosed with colorectal cancer in the last 5 years and have underwent or are currently undergoing treatment?

- Yes
- No

Have you been diagnosed with a second primary cancer (ex: breast, lung, prostate, etc.) in the last 5 years?

- Yes
- No

Pre-Survey Screening Questions for Caregivers

Do you currently reside in Canada?

- Yes
- No

Are you 18 years of age or older?

- Yes
- No

Have you provided care to someone diagnosed with colorectal cancer in the last 5 years who has underwent or is currently undergoing treatment?

- Yes
- No

Has the person you care for been diagnosed with a second primary cancer (e.g., breast, lung, prostate) within the past five years?

- Yes
- No

Pre-Survey Screening Questions for Healthcare Professionals

Do you currently practice in Canada?

- Yes
- No

Do you provide care to individuals who have been diagnosed with colorectal cancer?

- Yes
- No

Participant Information & Consent Form

Introduction

You are invited to participate in a research study. Before deciding, please review the process, risks, and benefits. Participation is voluntary—please take your time to make your decision.

Background and Purpose

- The purpose of this study is to explore patients', caregivers', and healthcare professionals' approach to and management of nutrition during colorectal cancer treatment. The study also aims to identify gaps in currently available oncology nutrition support resources.
- Malnutrition is a significant and often overlooked issue among cancer patients, made worse by both the disease and its treatments. It negatively impacts quality of life, increases treatment-related toxicities, and is a contributing factor in up to 20% of cancer-related deaths.

- Despite these challenges, there is no standardized approach to assessing and managing nutrition in oncology care.
- **385** participants from across Canada will be included in the study.

In this survey, you will be asked questions about your experience, knowledge, and challenges related to colorectal cancer and nutrition management. The information you provide is for research purposes only. The survey will take approximately 20 minutes to complete.

At the end of the survey, you have the option to leave your email to receive a lay summary of the aggregate study findings upon completion of the study. As a thank-you for your participation in the study, you will also have the option to leave your email to enter a draw to win a $25 Amazon card. There is no additional compensation for participating in the study.

Risks Related to Being in the Study

There are no known risks associated with contributing data as part of this study.

Benefits to Being in the Study

You will not benefit directly from taking part in this study.

Voluntary Participation

Participation in this study is entirely voluntary, and you may choose to stop at any point before submitting your survey responses.

Since all responses are collected anonymously, we are unable to identify individual submissions. As a result, once you have submitted your responses, we will not be able to remove your data upon request. By proceeding with the survey, you acknowledge that you understand and agree to these terms.

Confidentiality

Your participation in this study is completely anonymous and no personal identifying information will be collected.

If you choose to share your email address to enter the draw or the receive the summary of study findings, it will be stored separately from survey responses and will not be linked to your answers in any way. Email addresses will only be used for the specified purposes and deleted after study summaries have been sent and the gift card draw is completed.

“Study data" is information about you that is collected for the study, but that does not directly identify you. All study data collected will be stored securely and used only for research purposes. Survey responses will be analyzed in aggregate form, meaning that individual answers will not be singled out. The data will be stored on a secure, password-protected cloud service (SharePoint) and will only be accessible to authorized research team members and the William Osler Health System Review Board, which may access research records for ethics compliance. After the study has ended, data will be stored on Colorectal Cancer Canada’s SharePoint for seven years before secure destruction. The findings from this research may be shared in publications, presentations, or reports.

Privacy and Confidentiality Measures

The online survey is hosted by Purdie Pascoe, which stores data on SharePoint cloud services located in the UK. All data collected by Purdie Pascoe on behalf on the client (Colorectal Cancer Canada) is used solely in accordance with their expressly communicated intended purposes. Only authorized members of the client's team can access or download the data. All project files are securely deleted after 24 months, following their data retention policy.

Expenses Associated with Participating in the Study

There are no expenses associated with participating in the study.

Conflict of Interest

No conflict of interest to be declared.

Questions about the Study

If you have any questions, concerns or would like to speak to the study team for any reason, please email: Iris Karry at irisk@colorectalcancercanada.com.

If you have any questions about your rights as a research participant or have concerns about this study, call Dr. Herbert Brill, Chair of the William Osler Health System Research Ethics Board (REB) at 905-494-2120 ext. 50448. The REB is a group of people who oversee the ethical conduct of research studies. These people are not part of the study team. Everything that you discuss will be kept confidential.

A record of your virtual consent will be securely stored for reference.

By signing this consent form I agree that:

- This study has been explained to me and any questions I had have been answered.
- I know that my participation is voluntary and that I may leave the study at any time.
- I understand the requirements of participating in this research study
- I have been informed of the risks and benefits, if any, of participating in this research study
- I have been informed of the rights of research participants
- I have read each page of this form
- I authorize access to research study data as explained in this form

☐ I consent to participate in this study

☐ I do not consent to participate in this study

Participants who consent to the study will proceed to the survey questions; participants who do not consent to the study will be directed to exit the survey platform.

Patient Survey: Colorectal Cancer Nutritional Support

Instructions

This survey is divided into three sections. You will be asked to provide your demographic information, information related to your disease and nutritional status, and details about the nutritional counseling support you received during your cancer treatment.

Section 1. Demographic information

1. Which age group do you belong to?

- ☐ 18-30 years
- ☐ 31-40 years
- ☐ 41-50 years
- ☐ 51-60 years
- ☐ 61-70 years
- ☐ 71-79 years
- ☐ 80+ years
- ☐ Prefer not to answer

2. Please select the population group that applies to you:

- ☐ Arab
- ☐ Black (includes those of Black African descent and those who identify as descendants of Black African peoples)
- ☐ Chinese
- ☐ Filipino
- ☐ Japanese
- ☐ Korean
- ☐ Latin American
- ☐ Métis, First Nations, Inuit
- ☐ South Asian (e.g., East Indian, Pakistani, Sri Lankan)
- ☐ Southeast Asian (e.g., Vietnamese, Cambodian, Laotian, Thai)
- ☐ West Asian (e.g., Iranian, Afghan)
- ☐ White
- ☐ Other (Please specify: ______)
- ☐ Prefer not to answer

3. Select the term that best describe your gender identity:

- ☐ Woman
- ☐ Man
- ☐ Agender
- ☐ Bigender/Multigender
- ☐ Gender-fluid
- ☐ Gender Queer
- ☐ Nonbinary
- ☐ Questioning
- ☐ Transgender
- ☐ Two-Spirit
- ☐ A gender not specified above
- ☐ Prefer not to answer

4. What is your highest level of education completed?
- ☐ Less than high school diploma
- ☐ High school diploma or equivalent
- ☐ CÉGEP (Quebec)
- ☐ College certificate/diploma/trade college
- ☐ Bachelor's degree
- ☐ Master's degree
- ☐ Doctorate
- ☐ Other (Please specify: ______)
- ☐ Prefer not to answer

5. Select the province/territory where you currently reside:
- ☐ Alberta
- ☐ British Columbia
- ☐ Manitoba
- ☐ New Brunswick
- ☐ Newfoundland & Labrador
- ☐ Northwest Territories
- ☐ Nova Scotia
- ☐ Nunavut
- ☐ Ontario

- ☐ Prince Edward Island
- ☐ Quebec
- ☐ Saskatchewan
- ☐ Yukon
- ☐ Prefer not to answer

6. Which of the following options best represents the size of the population where you live?
   - ☐ Small population center, with a population of between 1,000 and 29,999
   - ☐ Medium population center, with a population of between 30,000 and 99,999
   - ☐ Large urban population center, consisting of a population of 100,000 and over
   - ☐ Rural area (RAs) (all territory lying outside population centers)
   - ☐ Prefer not to answer

Section 2. Disease-related information and nutritional status

7. What type of cancer have you been diagnosed with?
   - ☐ Colon
   - ☐ Rectal

8. What is the current stage of your cancer?
   - ☐ Stage I
   - ☐ Stage II
   - ☐ Stage III
   - ☐ Stage IV
   - ☐ Remission
   - ☐ I'm not sure

9. Which treatments for colorectal cancer have you received? (Select all that apply)
   - ☐ Chemotherapy
   - ☐ Immunotherapy
   - ☐ Targeted therapy
   - ☐ Radiation therapy
   - ☐ Surgery
   - ☐ Clinical trial treatments
   - ☐ Other (please specify: ______)

10. For how long did you receive (or are currently receiving) treatment?

- ☐ 1-3 months
- ☐ 4-6 months
- ☐ 7-12 months
- ☐ More than 12 months

11. Which of the following nutrition impact symptoms resulting from colorectal cancer or its treatment affect (or have affected) your daily life? (Select all that apply)

Nutrition impact symptoms *are symptoms that make it difficult for you to eat enough food.*

- ☐ Loss of appetite
- ☐ Nausea
- ☐ Vomiting
- ☐ Diarrhea
- ☐ Constipation
- ☐ Mouth sores
- ☐ Dry mouth
- ☐ Changes to how food tastes and smells
- ☐ Problems swallowing
- ☐ Indigestion
- ☐ Heartburn
- ☐ Feeling full too quickly after beginning to eat
- ☐ Pain
- ☐ Fatigue
- ☐ Other (please specify: _______)
- ☐ None of the above

12.

	While undergoing treatment for colorectal cancer, have you:			
		1	2	
		Yes	No	
(a)	Worried that you do not eat enough	☐	☐	
(b)	Had difficulty doing your usual activities because of weight loss	☐	☐	

(c)	Felt like your weight loss was out of control	☐	☐	
(d)	Received adequate information regarding weight loss	☐	☐	

13. Do you have any other conditions, such as diabetes, that cause nutrition impact symptoms? Please describe.

14. How do (or did) you manage nutrition impact symptoms (e.g. loss of appetite, changes to how food tastes smells, pain) that you experienced? (Select all that apply)

Nutrition impact symptoms *are symptoms that make it difficult for you to eat enough food.*

- ☐ I consulted with a provincially regulated nutrition professional such as a dietitian or nutritionist. *Note: A provincially regulated nutrition professional will have one of the following initials:* ***RD****,* ***RDN****,* ***PDt****, or* ***DtP, Dt.I, Dt.N.I*** *in French*
- ☐ I consulted with a non-provincially regulated nutrition professional, such as a Registered Holistic Nutritionist, Certified Nutritional Practitioner. *Note: A non-provincially regulated nutrition professional will have one of the following initials:* ***RONP, RNCP, ROHP, RHN, CNP***
- ☐ With nutritional supplements/meal replacements such as Ensure/Boost
- ☐ I consulted with a palliative/supportive care specialist
- ☐ I consulted with an endocrinologist
- ☐ I consulted with a naturopath
- ☐ I consulted with other healthcare professionals, please specify:
- ☐ With medications (prescription or over the counter), please specify:
- ☐ Through dietary changes, please specify:
- ☐ With physical rehabilitation or physical therapy/exercise
- ☐ Through meditation and mindfulness
- ☐ Through psychological counselling
- ☐ With support from family or friends
- ☐ I participated in support groups
- ☐ I consulted online communities/forums
- ☐ I consulted social media (e.g. influencers)
- ☐ I consulted websites, such as Canadian Cancer Society
- ☐ I consulted health care authorities (e.g., Health Canada)
- ☐ I consulted scientific journals
- ☐ I do or did not experience any nutritional concerns
- ☐ I took no special measure to manage my nutritional concerns
- ☐ Other, please specify:

15. How effective are (or were) the approaches that you selected in the previous question in addressing nutritional concernPs (e.g. weight loss, lack of appetite, difficulty eating due to side effects from treatment, possible nutritional deficiencies) that were associated with your treatment?

- ☐ Not effective at all
- ☐ Slightly effective
- ☐ Moderately effective
- ☐ Very effective
- ☐ Extremely effective

Please provide any comments on why you believe they were effective or not:

Section 3. Nutritional counselling received during and following your cancer treatment

16a. Since your cancer diagnosis, when has your nutritional status been discussed and assessed? (Select all that apply)

Nutritional status assessment: *A detailed check by healthcare professionals to understand your overall nutrition, identify signs of malnutrition, figure out the causes, and create a plan to improve your health. This could include an assessment of changes in weight, appetite and food intake, barriers to eating, possibly a physical exam focused on muscle loss.*

- ☐ At diagnosis
- ☐ Throughout treatment
- ☐ At the end of the treatment
- ☐ Never
- ☐ I don’t know/I don’t remember

16b. How many times did you receive nutritional support, such as a consultation with a dietitian, after your cancer diagnosis?

- ☐ None
- ☐ Once
- ☐ 2-3 times
- ☐ 4 times or more

17. After you received a cancer diagnosis, how long did you have to wait to connect with a dietitian for a first appointment?

- ☐ Less than 1 week
- ☐ 1-2 weeks
- ☐ 2-4 weeks
- ☐ 4-8 weeks
- ☐ More than 8 weeks

- ☐ I did not connect with a dietitian

18. In what format did you receive nutritional information during cancer treatment? (Select all that apply)

- ☐ Verbal guidance from a healthcare professional
- ☐ Printed handouts or brochures
- ☐ Digital resources (e.g., website, email, or app)
- ☐ Group classes or workshops
- ☐ Video or audio materials
- ☐ Other (please specify): _______

19. The professional nutritional support you received came from:

Professional nutritional support *refers to any expert guidance and interventions provided by trained healthcare professionals, such as dietitians or nutritionists.*

- ☐ The same institution where I am (or was) being treated
- ☐ External to the institution where I am (or was) being treated
- ☐ I did not receive professional nutritional support

20. Was the information you received from the professional on nutritional support understandable and relevant to your needs?

- ☐ Not understandable and relevant at all to my needs
- ☐ Slightly understandable and relevant
- ☐ Somewhat understandable and relevant
- ☐ Understandable and relevant
- ☐ Very understandable and relevant to my needs

Provide any additional comments:

21. How important is it that the nutritional information and recommendations that you receive be culturally sensitive and personalized according to your dietary preferences during or after treatment?

- ☐ Not important
- ☐ Slightly important
- ☐ Moderately important
- ☐ Very important
- ☐ Extremely important

22. From all the nutritional information and recommendations you received, which of them consider your dietary preferences and culturally preferred foods? (Select all that apply)

- ☐ Nutrition counseling by dietitians or nutritionists
- ☐ Support groups for cancer patients focusing on nutrition
- ☐ Workshops or classes on healthy eating for cancer patients
- ☐ Meal planning services
- ☐ Online resources or virtual support groups
- ☐ Home delivery of nutritious meals
- ☐ Financial assistance for nutritional support
- ☐ Community kitchens or cooking classes
- ☐ I did not receive nutritional information and recommendations
- ☐ Other (please specify: ______)

23. To what extent do the nutritional information and recommendations you received consider your dietary preferences and culturally preferred foods?

- ☐ Not at all
- ☐ Minimally
- ☐ Somewhat
- ☐ Largely
- ☐ Completely
- ☐ I did not receive nutritional information/recommendations

24. How would you rate your overall experience with the nutritional support services provided?

- ☐ Very poor
- ☐ Poor
- ☐ Neutral
- ☐ Good
- ☐ Excellent

25. What factors do you believe help or hinder your ability to maintain good nutritional habits? Please elaborate.

26. What changes or improvements would you suggest to enhance the quality of nutritional support services? Please elaborate.

Thank you for completing this survey and providing us with this information. Your participation is extremely valued and appreciated.

If you would like to receive a summary of the study findings upon its completion, and/or to enter a draw to win a $25 Amazon gift card, click Finish.

Caregiver Survey: Colorectal Cancer Nutritional Support

Instructions

This survey is divided into three sections. You will be asked to provide your demographic information, share your caregiving experience, and describe your role in providing nutritional support to the person with colorectal cancer under your care.

Section 1. Demographic information

1. What age group do you belong to?

- ☐ 18-30 years
- ☐ 31-40 years
- ☐ 41-50 years
- ☐ 51-60 years
- ☐ 61-70 years
- ☐ 71-79 years
- ☐ 80+ years
- ☐ Prefer not to answer

2. Please select the population group that applies to you:

- ☐ Arab
- ☐ Black (includes those of Black African descent and those who identify as descendants of Black African peoples)
- ☐ Chinese
- ☐ Filipino
- ☐ Japanese
- ☐ Korean
- ☐ Latin American
- ☐ Métis, First Nations, Inuit
- ☐ South Asian (e.g., East Indian, Pakistani, Sri Lankan)
- ☐ Southeast Asian (e.g., Vietnamese, Cambodian, Laotian, Thai)
- ☐ West Asian (e.g., Iranian, Afghan)
- ☐ White
- ☐ Other (Please specify: ______)
- ☐ Prefer not to answer

3. Select the term(s) that best describe your current gender identity:
   - ☐ Woman
   - ☐ Man
   - ☐ Agender
   - ☐ Bigender/Multigender
   - ☐ Gender-fluid
   - ☐ Gender Queer
   - ☐ Nonbinary
   - ☐ Questioning
   - ☐ Transgender
   - ☐ Two-Spirit
   - ☐ A gender not specified above
   - ☐ Prefer not to answer

4. What is your highest level of education completed?
   - ☐ Less than high school diploma
   - ☐ High school diploma or equivalent
   - ☐ CÉGEP (Quebec)
   - ☐ College certificate/diploma/trade college
   - ☐ Bachelor’s degree
   - ☐ Master’s degree
   - ☐ Doctorate
   - ☐ Other (Please specify: ______)
   - ☐ Prefer not to answer

5. Select the province/territory where you currently reside:
   - ☐ Alberta
   - ☐ British Columbia
   - ☐ Manitoba
   - ☐ New Brunswick
   - ☐ Newfoundland & Labrador
   - ☐ Northwest Territories
   - ☐ Nova Scotia

- ☐ Nunavut
- ☐ Ontario
- ☐ Prince Edward Island
- ☐ Quebec
- ☐ Saskatchewan
- ☐ Yukon
- ☐ Prefer not to answer

6. Which of the following options best represents the size of the population where you live?
- ☐ Small population center, with a population of between 1,000 and 29,999
- ☐ Medium population center, with a population of between 30,000 and 99,999
- ☐ Large urban population center, consisting of a population of 100,000 and over
- ☐ Rural area (RAs) (all territory lying outside population centers)
- ☐ Prefer not to answer

7. The person with colorectal cancer whose care I am supporting is my:
- ☐ Spouse/Partner
- ☐ Parent
- ☐ Child
- ☐ Sibling
- ☐ Friend
- ☐ Other (Please specify: ______)
- ☐ Prefer not to answer

8. How long have you been providing care to the person living with colorectal cancer?
- ☐ Less than 6 months
- ☐ 6-12 months
- ☐ 1-2 years
- ☐ More than 2 years

Section 2. Caregiving Experience

9. What types of care do you provide as a caregiver? (Select all that apply)
- ☐ Personal care (e.g., bathing, dressing)

- ☐ Medication management
- ☐ Grocery shopping
- ☐ Meal preparation and planning
- ☐ Transportation to medical appointments
- ☐ Emotional support
- ☐ Other (Please specify: _______)

Section 3. Caregiving experience providing nutritional support to an individual living with CRC

10. Which of the following nutrition impact symptoms resulting from cancer and treatment affect (or have affected) the person you care for during treatment? (Select all that apply)

Nutrition impact symptoms *are symptoms that make it difficult for a person to eat enough food.*

- ☐ Loss of appetite
- ☐ Nausea
- ☐ Vomiting
- ☐ Diarrhea
- ☐ Constipation
- ☐ Mouth sores
- ☐ Dry mouth
- ☐ Indigestion
- ☐ Heartburn
- ☐ Changes to how food tastes and smells
- ☐ Problems swallowing
- ☐ Feeling full too quickly after beginning to eat
- ☐ Pain
- ☐ Fatigue
- ☐ Other (please specify: _______)
- ☐ None of the above

11.

	While undergoing treatment for colorectal cancer, did the person you care for:		
	1	2	
	Yes	No	

(a)	Worry that they do not eat enough	☐	☐	
(b)	Have difficulty doing their usual activities because of weight loss	☐	☐	
(c)	Feel like their weight loss was out of control	☐	☐	
(d)	Receive adequate information regarding weight loss	☐	☐	

12. Does the person you care for have any other conditions, such as diabetes, that cause nutrition impact symptoms? Please describe.

13. How were the nutritional concerns and nutrition impact symptoms of the person you care for managed during treatment? (Select all that apply).

***Nutrition impact symptoms** are symptoms that make it difficult for a person to eat enough food.*

- ☐ Through consultation with a provincially regulated nutrition professional such as a dietitian or nutritionist. *Note: A provincially regulated nutrition professional will have one of the following initials:* ***RD****,* ***PDt****, or* ***DtP*** *in French*

- ☐ Through consultation with a non-provincially regulated nutrition professional, such as a Registered Holistic Nutritionist, Certified Nutritional Practitioner. *Note: A non-provincially regulated nutrition professional will have one of the following initials:* ***RONP, RNCP, ROHP, RHN, CNP***

- ☐ With nutritional supplements/meal replacements such as Ensure/Boost
- ☐ Through consultation with a palliative/supportive care specialist
- ☐ Through consultation with an endocrinologist
- ☐ Through consultation with a naturopath
- ☐ With medications (prescription or over the counter), please specify:
- ☐ Through dietary changes, please specify:
- ☐ With physical rehabilitation or physical therapy/exercise
- ☐ Through meditation and mindfulness
- ☐ Through psychological counselling
- ☐ With support from family or friends
- ☐ Through support groups
- ☐ By consulting online communities/forums
- ☐ By consulting social media (e.g. influencers)
- ☐ By consulting websites, such as Canadian Cancer Society
- ☐ By consulting healthcare authorities (e.g. Health Canada)
- ☐ By consulting scientific journals
- ☐ I can or could not manage the side effects they experienced
- ☐ They do or did not experience any nutritional concerns

- ☐ No special measures were taken to manage nutritional concerns
- ☐ Other, please specify:

13. How confident are you in your ability to help the person you care for manage treatment side effects that may arise such as tiredness, loss of appetite, nausea, or a changing appetite?

- ☐ Not confident at all
- ☐ Slightly confident
- ☐ Moderately confident
- ☐ Very confident
- ☐ Extremely confident

14. How frequently do you need help or guidance in dealing with the eating habits of the person you care for?

- ☐ Always
- ☐ Often
- ☐ Sometimes
- ☐ Rarely
- ☐ Never

15. As a caregiver, what type of nutritional resources have been available to you? (Select all that apply)

- ☐ Guidance from a dietitian
- ☐ Guidance from other healthcare professionals, please specify:
- ☐ Printed handouts or brochures
- ☐ Digital resources (e.g., website, email, or app)
- ☐ Group classes or workshops
- ☐ Video or audio materials
- ☐ Other (please specify): _______

16. How easy was it to access these resources?

- ☐ Very easy
- ☐ Somewhat easy
- ☐ Neither easy not difficult
- ☐ Somewhat difficult
- ☐ Very difficult

17. How effective are (or were) the nutritional guidance and recommendations in addressing treatment side effects?

- ☐ Very ineffective
- ☐ Somewhat ineffective
- ☐ Neutral
- ☐ Somewhat effective
- ☐ Very effective

18. Did the resources consider foods and eating habits preferred in your culture or avoided for cultural reasons? Please explain.

19. To what extent do you feel that the nutritional support you received has positively impacted the health and recovery of the person you care for?

- ☐ No impact
- ☐ Minor impact
- ☐ Moderate impact
- ☐ Significant impact
- ☐ Very significant impact
- ☐ Not applicable

20. How would you rate your overall experience with the nutritional support services provided to you as a caregiver?

- ☐ Very poor
- ☐ Poor
- ☐ Neutral
- ☐ Good
- ☐ Excellent

21. What improvements or additional resources would help you better manage the nutritional needs of the person you care for and support your well-being as a caregiver? (Select all that apply)

- ☐ More comprehensive nutritional guidelines
- ☐ Easier access to dietitians or nutritionists
- ☐ Support groups or counseling for caregivers
- ☐ Resources for managing stress and emotional well-being
- ☐ Better access to nutritious and suitable foods
- ☐ Training on handling nutrition-related side effects from treatment
- ☐ Other (please specify): ___________

22. What advice would you give to someone caring for a person undergoing cancer treatment regarding managing the patient's nutrition? Please explain.

23. Do you have any additional suggestions or comments on how nutritional support services and resources can be improved to better meet the needs of patients and caregivers? Please elaborate.

Thank you for completing this survey and providing us with this information. Your participation is extremely valued and appreciated.

If you would like to receive a summary of the study findings upon its completion, and/or to enter a draw to win a $25 Amazon gift card, click Finish.

Healthcare Provider Survey: Colorectal Cancer Nutritional Support

Instructions

This survey is divided into five sections. You will be asked to provide your demographic information, share your experience in treating colorectal cancer patients, and describe how you address their nutritional needs and preferences.

Section 1. Demographic information

1. What is your professional role?

- ☐ Dietitian
- ☐ Nutritionist
- ☐ Pivot nurse/nurse navigator
- ☐ Family Practitioner
- ☐ Oncologist (Medical, Radiation, Surgical)
- ☐ Physical Therapist
- ☐ Occupational Therapist
- ☐ Palliative/supportive care specialist
- ☐ Other (please specify: ______)

2. What types of training have you received specifically related to nutrition and cancer care? (Select all that apply)

- ☐ Formal education (e.g., university degree and courses)
- ☐ Workshops or seminars
- ☐ Online courses or webinars
- ☐ Other (please specify: ______)
- ☐ None

3. Which province or territory do you currently practice in?

- ☐ Alberta
- ☐ British Columbia
- ☐ Manitoba
- ☐ New Brunswick
- ☐ Newfoundland & Labrador
- ☐ Northwest Territories
- ☐ Nova Scotia
- ☐ Nunavut
- ☐ Ontario
- ☐ Prince Edward Island
- ☐ Quebec
- ☐ Saskatchewan
- ☐ Yukon
- ☐ Prefer not to answer

4. What type of healthcare facility do you practice in?

- ☐ Academic hospital
- ☐ Community hospital
- ☐ Group/family practice
- ☐ Solo office practice
- ☐ Other (please specify: ______)

5. Which of the following options best represents the size of the population where you practice?

- ☐ Small population center, with a population of between 1,000 and 29,999
- ☐ Medium population center, with a population of between 30,000 and 99,999
- ☐ Large urban population center, consisting of a population of 100,000 and over
- ☐ Rural area (RAs) (all territory lying outside population centers)
- ☐ Prefer not to answer

Section 2. Treating colorectal cancer patients

6. What stages do you most frequently encounter in your patients with colorectal cancer? (Select all that apply)

- ☐ Stage I
- ☐ Stage II

- ☐ Stage III
- ☐ Stage IV
- ☐ In remission

7. Which of the following treatments have the greatest impact on patients' nutritional status? (Select all that apply)

- ☐ Chemotherapy
- ☐ Immunotherapy
- ☐ Targeted therapy
- ☐ Radiation therapy
- ☐ Surgery
- ☐ Other (please specify: ______)

8. For how long do the patients under your care typically receive cancer treatment?

- ☐ 1-3 months
- ☐ 4-6 months
- ☐ 7-12 months
- ☐ More than 12 months
- ☐ I don't know

9. Which of the following nutrition impact symptoms resulting from the cancer and treatment are commonly reported by colorectal cancer patients?

Nutrition impact symptoms *are symptoms that make it difficult for your patient to eat enough food.*

- ☐ Loss of appetite
- ☐ Nausea
- ☐ Vomiting
- ☐ Diarrhea
- ☐ Constipation
- ☐ Mouth sores
- ☐ Dry mouth
- ☐ Indigestion
- ☐ Heartburn
- ☐ Changes to how food tastes and smells
- ☐ Problems swallowing
- ☐ Feeling full too quickly after beginning to eat

- ☐ Pain
- ☐ Fatigue
- ☐ Other (please specify: _______)
- ☐ None of the above

Section 3. Addressing nutritional needs and preferences in colorectal cancer patients

10. How important do you think professional nutrition support is for patients during cancer treatment?

- ☐ Not at all Important
- ☐ Slightly Important
- ☐ Moderately Important
- ☐ Very Important
- ☐ Extremely Important

11. Does your center systematically screen and assess patients for risk of malnutrition? If yes, how? e.g. through use of a validated screening and assessment tool. Please elaborate.

12. What happens for patients that screen positive? For example, are they referred to a dietitian? Please describe.

13.

What proportion of the patients you see in your practice experience:		%
(a)	Sarcopenia<br>*A condition characterized by the progressive loss of skeletal muscle mass, strength, and function, primarily associated with aging but can be accelerated by chronic diseases such as cancer, poor nutrition, and inactivity.*	
(b)	Cachexia<br>*Also called wasting syndrome or anorexia cachexia syndrome. It is a complex problem that is more than a loss of appetite. It involves changes in the way the body uses proteins, carbohydrates, and fat. Patients may also burn calories faster than usual.*	

14. When patients experience general malnutrition and/or cachexia during cancer treatment, which healthcare professionals are typically involved in their care?

- ☐ Dietitian
- ☐ Nutritionist
- ☐ Physical therapist

- ☐ Occupational therapist
- ☐ Social worker
- ☐ Mental health professional
- ☐ Palliative/supportive care specialist
- ☐ Endocrinologist
- ☐ Other, please specify:

Please provide any additional comments.

15. What types of interventions are used to manage cachexia and general malnutrition/risk for malnutrition in patients under your care? (Select all that apply)

- ☐ Referral to a dietitian
- ☐ Medications, please specify:
- ☐ Dietary changes, please specify:
- ☐ Nutritional supplements/meal replacements such as Ensure/Boost
- ☐ Physical rehabilitation or physical therapy
- ☐ Meditation and mindfulness
- ☐ Psychological counselling
  - ☐ Other (please specify: ______)
  - ☐ None

16. At your healthcare institution, are nutrition or dietitian services provided on an in-patient basis, an out-patient basis, or both?

  - ☐ In-patient only
  - ☐ Out-patient only
  - ☐ Both in-patient and out-patient
  - ☐ Not sure

17. How long does it typically take for colorectal cancer patients under your care to connect with a dietitian?

  - ☐ Less than 1 week
  - ☐ 1-2 weeks
  - ☐ 2-4 weeks
  - ☐ 4 weeks or more
  - ☐ Most of my patients do not connect with a dietitian

18. In what formats are initial nutritional recommendations usually explained to patients? (Select all that apply)

- ☐ Printed handouts or brochures
- ☐ Digital resources (e.g., website, email, or app)
- ☐ Verbal guidance from a healthcare provider
- ☐ Group classes or workshops
- ☐ Video or audio materials
- ☐ I’m not sure
- ☐ Other (please specify): _______

19. What types of nutritional support do CRC patients typically access? (Select all that apply)

- ☐ Nutrition counseling by dietitians or nutritionists
- ☐ Support groups for cancer patients focusing on nutrition
- ☐ Workshops or classes on healthy eating for cancer patients
- ☐ Meal planning services
- ☐ Online resources or virtual support groups
- ☐ Home delivery of nutritious meals
- ☐ Financial assistance for nutritional support
- ☐ Community kitchens or cooking classes
- ☐ I’m not sure
- ☐ Other (please specify: ______)

20. From your experience, how would you rate the availability of resources which provide nutritional guidance to colorectal cancer patients?

- ☐ Very poor
- ☐ Poor
- ☐ Moderate
- ☐ Good
- ☐ Excellent

Section 4. Nutrition-assessment practices

21. At what stages of treatment do you collaborate with members of the cancer care team to support the patient’s nutritional needs?

- ☐ At diagnosis
- ☐ At every appointment

- ☐ Monthly
- ☐ Every 3 months
- ☐ Every 6 months
- ☐ Other (please specify: ______)

22. How often do you or your team members conduct the following assessments for cancer patients? (Please select the typical frequency.)

Weight monitoring

- ☐ At every appointment
- ☐ Monthly
- ☐ Every 3 months
- ☐ Post-treatment
- ☐ Other (please specify: ______)
- ☐ N/A

Objective and quantitative assessment of nutritional intake (for patients with abnormal screening)

- ☐ At every appointment
- ☐ Monthly
- ☐ Every 3 months
- ☐ Post-treatment

☐ Other (please specify: ______)

- ☐ N/A

Assessment of malnutrition-related symptoms (for patients with abnormal screening)

- ☐ At every appointment
- ☐ Monthly
- ☐ Every 3 months
- ☐ Post-treatment

☐ Other (please specify: ______)

- ☐ N/A

Assessment of muscle mass (for patients with abnormal screening)

- ☐ At every appointment
- ☐ Monthly
- ☐ Every 3 months
- ☐ Post-treatment

-☐ Other (please specify: ______)

- ☐ N/A

Assessment of physical performance (for patients with abnormal screening)

- ☐ At every appointment
- ☐ Monthly
- ☐ Every 3 months
- ☐ Post-treatment

☐ Other (please specify: ______)

- ☐ N/A

Assessment of the degree of systemic inflammation, e.g. measurement of C-reactive protein (for patients with abnormal screening)

- ☐ At every appointment
- ☐ Monthly
- ☐ Every 3 months
- ☐ Post-treatment

☐ Other (please specify: ______)

- ☐ N/A

23. How do you assess patient compliance with nutritional recommendations? (Select all that apply)

- ☐ Patient self-reports
- ☐ Review of dietary logs
- ☐ Medical outcomes
- ☐ Information from caregivers
- ☐ Other (please specify: ______)

24. In your opinion, how well do current nutritional resources address patients' dietary preferences and cultural considerations?

- ☐ Very poorly
- ☐ Poorly
- ☐ Somewhat
- ☐ Well
- ☐ Very well

Section 5: Feedback and Improvement

25. From your experience, what are the barriers to patients receiving timely nutrition support at your institution?

26. What changes or improvements would you suggest to enhance the quality of nutritional support services and resources? Please elaborate.

Thank you for completing this survey and providing us with this information. Your participation is extremely valued and appreciated.

If you would like to receive a summary of the study findings upon its completion, and/or to enter a draw to win a $25 Amazon gift card, click Finish.
