# Supplementary material for: Nutrition Care Practices in Colorectal Cancer: A National Survey of Patients, Caregivers, and Healthcare Professionals in Canada
Source: Curr Oncol. 2026 Sep 10;33(9):547. doi: 10.3390/curroncol33090547 (PMC13605743; doi:10.3390/curroncol33090547)
Supplement: Supplementary file 1 [file curroncol-33-00547-s001.zip › Tables S1-S5.pdf]

Table S1. Patient Respondent Demographic (n=121)

Characteristics	n (%)
**Age group**	
18-30 years	12 (9.9)
31-40 years	32 (26.4)
41-50 years	30 (24.8)
51-60 years	21 (17.4)
61-70 years	18 (14.9)
71-79 years	8 (6.6)
80+ years	0 (0.0)
**Ethnicity**	
Arab	2 (1.7)
Black	11 (9.1)
Chinese	5 (4.1)
Filipino	2 (1.7)
Japanese	1 (0.8)
Korean	0 (0.0)
Latin American	3 (2.5)
Métis, First Nations, Inuit	0 (0.0)
South Asian	1 (0.8)
Southeast Asian	2 (1.7)
West Asian	0 (0.0)
White	93 (76.9)
Prefer not to answer	1 (0.8)
**Gender**	
Woman	65 (53.7)
Man	54 (44.6)
Bigender/Multigender	0 (0.0)
Gender-fluid	1 (0.8)
Other listed identities*	0 (0.0)
Prefer not to answer	1 (0.8)
**Education**	
Less than high school diploma	1 (0.8)
High school diploma or equivalent	14 (11.6)
CÉGEP (Quebec)	2 (1.7)
College certificate/diploma/trade college	30 (24.8)
Bachelor’s degree	46 (38.0)
Master’s degree	20 (16.5)
Doctorate	5 (4.1)
Other (Please specify)	0 (0.0)
Prefer not to answer	3 (2.5)
**Province/Territory**	
Alberta	14 (11.6)
British Columbia	21 (17.4)
Manitoba	6 (5.0)
New Brunswick	5 (4.1)
Newfoundland & Labrador	4 (3.3)
Northwest Territories	3 (2.5)
Nova Scotia	10 (8.3)
Nunavut	2 (1.7)
Ontario	30 (24.8)
Prince Edward Island	2 (1.7)
Quebec	12 (9.9)
Saskatchewan	1 (0.8)
Yukon	0 (0.0)
Prefer not to answer	11 (9.1)
**Size of Population**	
Small population center	16 (13.2)
Medium population center	34 (28.1)

Large urban population center	65 (53.7)
Rural area	5 (4.1)
Prefer not to answer	1 (0.8)
**Cancer Type**	
Colon	38 (31.4)
Rectal	83 (68.6)
**Cancer Stage**	
Stage I	18 (14.9)
Stage II	33 (27.3)
Stage III	24 (19.8)
Stage IV	25 (20.7)
Remission	17 (14.0)
I'm not sure	4 (3.3)
**Cancer Treatment**	
Chemotherapy	88 (72.7)
Immunotherapy	16 (12.2)
Targeted Therapy	25 (20.7)
Radiation	43 (35.5)
Surgery	80 (66.1)
Clinical trial treatments	6 (5)

n: sample size; *Other listed identities option included agender, gender queer, nonbinary, questioning, transgender, two-spirit, and "a gender not specified above".

Table S2. Caregiver Respondent Demographic (n=45)

Characteristics	n (%)
**Age group**	
18-30 years	8 (17.8)
31-40 years	20 (44.4)
41-50 years	9 (20.0)
51-60 years	3 (6.7)
61-70 years	4 (8.9)
71-79 years	1 (2.2)
80+ years	0 (0.0)
**Ethnicity**	
Arab	2 (4.4)
Black	8 (17.8)
Chinese	5 (11.1)
Filipino	1 (2.2)
Japanese	1 (2.2)
Korean	1 (2.2)
Latin American	3 (6.7)
Métis, First Nations, Inuit	2 (4.4)
South Asian	0 (0.0)
Southeast Asian	2 (4.4)
West Asian	0 (0.0)
White	18 (40.0)
Prefer not to answer	2 (4.4)
**Gender**	
Woman	26 (57.8)
Man	17 (37.8)
Bigender/Multigender	1 (2.2)
Gender-fluid	1 (2.2)
Other listed identities*	0 (0.0)
Prefer not to answer	0 (0.0)
**Education**	
Less than high school diploma	0 (0.0)
High school diploma or equivalent	9 (20.0)
CÉGEP (Quebec)	1 (2.2)

College certificate/diploma/trade college	15 (33.3)
Bachelor's degree	14 (31.1)
Master's degree	3 (6.7)
Doctorate	3 (6.7)
Other (Please specify)	0 (0.0)
Prefer not to answer	0 (0.0)
**Province/Territory**	
Alberta	8 (17.8)
British Columbia	3 (6.7)
Manitoba	4 (8.9)
New Brunswick	2 (4.4)
Newfoundland & Labrador	5 (11.1)
Northwest Territories	3 (6.7)
Nova Scotia	4 (8.9)
Nunavut	1 (2.2)
Ontario	9 (20.0)
Prince Edward Island	0 (0.0)
Quebec	4 (8.9)
Saskatchewan	1 (2.2)
Yukon	0 (0.0)
Prefer not to answer	1 (2.2)
**Size of Population**	
Small population center	4 (8.9)
Medium population center	23 (51.1)
Large urban population center	14 (31.1)
Rural area	3 (6.7)
Prefer not to answer	1 (2.2)
**Care Recipient**	
Spouse/Partner	12 (26.7)
Friend	12 (26.7)
Child	7 (15.6)
Sibling	5 (11.1)
Parent	3 (6.7)
Other	2 (4.4)
Prefer not to answer	4 (8.9)
**Caregiving Period**	
Less than 6 months	9 (20.0)
6-12 months	15 (33.3)
1-2 years	12 (26.7)
More than 2 years	9 (20.0)

n: sample size; *Other listed identities option included agender, gender queer, nonbinary, questioning, transgender, two-spirit, and "a gender not specified above".

Table S3. Healthcare Professional Respondent Demographic (n=77)

Characteristics	n (%)
**Professional Role**	
Dietitian	16 (20.8)
Family Practitioner	3 (3.9)
Nutritionist	14 (18.2)
Occupational Therapist	7 (9.1)
Oncologist (Medical, Radiation, Surgical)	8 (10.4)
Palliative/supportive care specialist	0 (0.0)
Physical Therapist	7 (9.1)
Pivot nurse/nurse navigator	13 (16.9)
Other*	9 (11.7)
*Other roles included Gastroenterologist and specialized nursing roles, including wound, ostomy, continence; surgical, oncology, and cachexia care.	
**Training related to nutrition and cancer care**	

Formal education (e.g., university degree and courses)	38 (49.4)
Workshops or seminars	50 (64.9)
Online courses or webinars	45 (58.4)
Other*	5 (6.5)
None	2 (2.6)
**Province/Territory**	
Alberta	16 (20.8)
British Columbia	8 (10.4)
Manitoba	6 (7.8)
New Brunswick	6 (7.8)
Newfoundland & Labrador	8 (10.4)
Northwest Territories	4 (5.2)
Nova Scotia	4 (5.2)
Nunavut	0 (0.0)
Ontario	12 (15.6)
Prince Edward Island	0 (0.0)
Quebec	7 (9.1)
Saskatchewan	6 (7.8)
Yukon	0 (0.0)
Prefer not to answer	0 (0.0)
**Size of Population**	
Small population center	6 (7.8)
Medium population center	25 (32.5)
Large urban population center	44 (57.1)
Rural area	2 (2.6)
Prefer not to answer	0 (0.0)
**Healthcare facility practiced in**	
Academic hospital	41 (53.2)
Community hospital	27 (35.1)
Group/family practice	5 (6.5)
Solo office practice	3 (3.9)
Other	1 (1.3)

n: sample size; *Other listed training included: self-directed research, reading textbook and articles, continued learning, and professional conference

Table S4. Patient Perceived Effectiveness of Nutrition Support and Information and Unmet Needs in Managing Nutrition Impact Symptoms (n=121)

Variable	n (%)
**How effective are (or were) the approaches you selected in managing nutrition impact symptoms in addressing nutritional concerns (e.g. weight loss, lack of appetite, difficulty eating due to side effects from treatment, possible nutritional deficiencies) that were associated with your treatment?**	
Not effective at all	5 (4.1)
Slightly effective	29 (24.0)
Moderately effective	35 (28.9)
Very effective	34 (28.1)
Extremely effective	18 (14.9)
**Was the information you received from the professional on nutritional support understandable and relevant to your needs?**	
Not understandable and relevant at all to my needs	3 (2.5)
Slightly understandable and relevant	13 (10.7)
Somewhat understandable and relevant	29 (24.0)
Understandable and relevant	30 (24.8)
Very understandable and relevant to my needs	29 (24.0)
I did not receive professional nutrition support	17 (14.0)
**How would you rate your overall experience with the nutritional support services provided?**	
Very poor	9 (7.4)
Poor	13 (10.7)

Neutral	40 (33.1)
Good	40 (33.1)
Excellent	19 (15.7)

n: sample size

Table S5. Caregiver Perceived Effectiveness of Nutrition Support and Information and Unmet Needs in Managing Nutrition Impact Symptoms (n=45)

Variable	n (%)
**How easy was it to access nutrition support and information?**	
Very easy	9 (20.0)
Somewhat easy	15 (33.3)
Neither easy nor difficult	15 (33.3)
Somewhat difficult	6 (13.3)
Very difficult	0 (0.0)
**How effective are (or were) the nutritional guidance and recommendations in addressing treatment side effects?**	
Very ineffective	1 (2.2)
Somewhat ineffective	3 (6.7)
Neutral	10 (22.2)
Somewhat effective	20 (44.4)
Very effective	11 (24.4)
**To what extent do you feel that the nutritional support you received has positively impacted the health and recovery of the person you care for?**	
No impact	0 (0.0)
Minor impact	6 (13.3)
Moderate impact	16 (35.6)
Significant impact	11 (24.4)
Very significant impact	12 (26.7)
Not applicable	0 (0.0)
**How would you rate your overall experience with the nutritional support services provided to you as a caregiver?**	
Very poor	0 (0.0)
Poor	4 (8.9)
Neutral	16 (35.6)
Good	18 (40.0)
Excellent	7 (15.6)

n: sample size
